# Japanese Encephalitis Surveillance and Immunization — Asia and Western Pacific Regions, 2016

**DOI:** 10.15585/mmwr.mm6622a3

**Published:** 2017-06-09

**Authors:** James D. Heffelfinger, Xi Li, Nyambat Batmunkh, Varja Grabovac, Sergey Diorditsa, Jayantha B. Liyanage, Sirima Pattamadilok, Sunil Bahl, Kirsten S. Vannice, Terri B. Hyde, Susan Y. Chu, Kimberley K. Fox, Susan L. Hills, Anthony A. Marfin

**Affiliations:** ^1^World Health Organization, Regional Office for the Western Pacific Region, Manila, Philippines; ^2^World Health Organization, Regional Office for South-East Asia, New Delhi, India; ^3^Immunizations, Vaccines and Biologicals, World Health Organization, Geneva, Switzerland; ^4^Global Immunization Division, Center for Global Health, CDC; ^5^Division of Vector-Borne Diseases, National Center for Emerging and Zoonotic Infectious Diseases, CDC; ^6^PATH, Seattle, Washington.

Japanese encephalitis (JE) virus is the most important vaccine-preventable cause of encephalitis in the Asia-Pacific region. The World Health Organization (WHO) recommends integration of JE vaccination into national immunization schedules in all areas where the disease is a public health priority (*1*). This report updates a previous summary of JE surveillance and immunization programs in Asia and the Western Pacific in 2012 (*2*). Since 2012, funding for JE immunization has become available through the GAVI Alliance, three JE vaccines have been WHO-prequalified,* and an updated WHO JE vaccine position paper providing guidance on JE vaccines and vaccination strategies has been published (*1*). Data for this report were obtained from a survey of JE surveillance and immunization practices administered to health officials in countries with JE virus transmission risk, the 2015 WHO/United Nations Children's Fund Joint Reporting Form on Immunization, notes and reports from JE meetings held during 2014–2016, published literature, and websites. In 2016, 22 (92%) of 24 countries with JE virus transmission risk conducted JE surveillance, an increase from 18 (75%) countries in 2012, and 12 (50%) countries had a JE immunization program, compared with 11 (46%) countries in 2012. Strengthened JE surveillance, continued commitment, and adequate resources for JE vaccination should help maintain progress toward prevention and control of JE.

JE is a mosquito-borne disease that is a leading cause of encephalitis in Asia (*1*). More than 3 billion persons live in 24 countries that have JE virus transmission risk areas (Figure) (*1*,*3*). The majority (75%) of JE cases occur in children aged <15 years (*3*). Although most JE cases are asymptomatic, the case fatality rate among patients with encephalitis approaches 30%, and approximately 30%–50% of survivors have long-term neurologic sequelae (*4*). Vaccination is the cornerstone of JE control and prevention measures (*1*). A 2011 systematic review of JE disease burden estimated that approximately 68,000 cases occur globally each year; only about 10% of these cases are reported to WHO (*3*).

**FIGURE:**
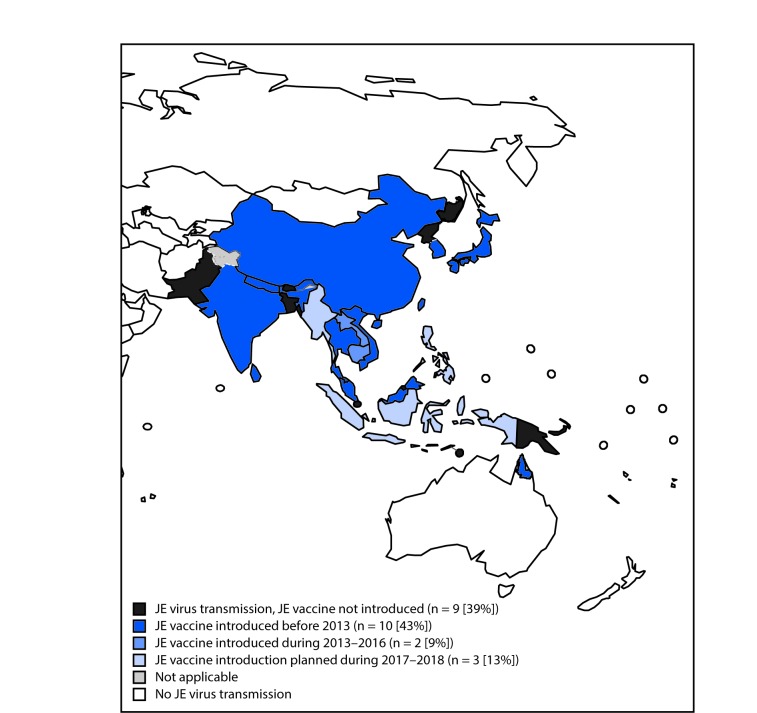
Areas with risk for Japanese encephalitis (JE) virus transmission and JE vaccine introduction* — 24 countries in Asia and the Western Pacific Region,^†^^,^^§^ 2016 **Source:** World Health Organization (WHO)/Immunization Vaccines and Biologicals database; May 12, 2017. * Singapore made a decision not to introduce JE vaccine because only rare, sporadic human cases are reported in the country. ^†^ The boundaries and names shown and the designations used on this map do not imply the expression of any opinion whatsoever on the part of the WHO concerning the legal status of any country, territory, city, or area or of its authorities, or concerning the delimitation of its frontiers or boundaries. Dotted lines on maps represent approximate border lines for which there might not yet be full agreement. ^§^ JE vaccine introduction in Indonesia will be limited to Bali.

Information on JE surveillance and immunization programs was obtained from several sources. Health officials from 18 WHO countries with endemic JE who attended the 7th Biregional Meeting on Prevention and Control of JE in 2016 were surveyed; abbreviated surveys^†^ were sent to health officials from six additional countries with endemic JE. Unpublished 2016 meeting notes, 2015 Joint Reporting Form on Immunization^§^ reports (*5*), the 2014 report of the 6th Biregional Meeting on Prevention and Control of JE (*6*), unpublished meeting notes from the 2015 Biregional Workshop on Strengthening the Capacity of the JE Laboratory Network in the WHO South-East Asian and Western Pacific Regions, and published literature and Ministry of Health websites served as additional data sources. Information collected about surveillance programs included a description of the surveillance system; case definitions used; age groups under surveillance; availability of diagnostic testing; and 2015 case numbers. Information collected on immunization programs included whether the country had an established JE immunization program, age of the first dose in the immunization schedule, and types of vaccines used.

## Surveillance Programs

Representatives from all 24 countries with JE virus transmission risk completed the surveys.^¶^ In 2016, 22 (92%) of the 24 countries conducted JE surveillance. Fourteen (58%) countries conducted national JE surveillance, two (8%) conducted subnational surveillance in all JE risk areas, and 11 (46%) conducted sentinel surveillance (including five countries that also conducted surveillance nationally or in all risk areas) (Table 1). Among 11 countries with sentinel surveillance, the median number of sentinel sites was eight (range = 1–223). JE case definitions were used in 22 (92%) countries. Twelve (50%) countries used the WHO acute encephalitis syndrome (AES) case definition (*7*), four (17%) used an acute meningoencephalitis syndrome (AMES) case definition,** three (12%) used AES or AMES case definitions in different settings, and three (12%) used country-specified case definitions. All countries with JE surveillance reported that some or most suspected cases were confirmed using JE-specific diagnostic testing of serum or cerebrospinal fluid (CSF) or both.

**TABLE 1:** Characteristics of Japanese encephalitis (JE) surveillance in countries with JE virus transmission risk, 2016

Country	JE surveillance program (no. sentinel sites)	Case definition used	Integration of encephalitis and meningitis surveillance	Age groups under surveillance	Laboratory confirmation of suspected cases	CSF tested*	Serum tested*
Australia^†^	All risk areas^§^	Other^¶^	No	All	Yes	Most	Most
Bangladesh	Sentinel (4)	AMES	No	All	Yes	Most	Most
Bhutan	Sentinel (5)	WHO AES	No	<15 yrs	Yes	Some	Most
Brunei	National	WHO AES	Yes	All	Yes	No	Most
Burma	National	WHO AES	Yes	All	Yes	Some	Most
Cambodia	Sentinel (6)	AMES	No	<15 yrs	Yes	Most	Most
China	National and sentinel (27)	WHO AES (national); AMES (sentinel)	Yes**	All	Yes	Most	Most
Taiwan	All areas	Other^††^	NA	All	Yes	Yes^§§^	Yes^§§^
India	All risk areas and sentinel (223)	WHO AES	No	All	Yes	Most	Most
Indonesia	Sentinel (34)	WHO AES	No	All	Yes	No	Most
Japan	National	Other^¶¶^	No	All	Yes	Yes^§§^	Yes^§§^
Laos	National and sentinel (3)	AMES (national); WHO, AES, AMES (sentinel)	Yes***	All	Yes	Most	Most
Malaysia	National	Other^†††^	No	All	Yes	Most	Most
Nepal	National	WHO AES	No	All	Yes	Most	Some
North Korea	National	AMES	Yes	<15 yrs	Yes	Yes^§§§^	Yes^§§§^
Pakistan	None	—	—	—	—	—	—
Papua New Guinea	Sentinel (1)	WHO AES	No	<15 yrs	Yes	Most	Most
Philippines	Sentinel (9)	AMES	Yes	All	Yes	Most	Most
Russia^†^	None	—	—	—	—	—	—
Singapore	National	WHO AES	No	All	Yes	Most	Most
South Korea	National	WHO AES	No	All	Yes	Most	Most
Sri Lanka	National	WHO AES	No	All	Yes	Most	Some
Thailand	National and sentinel (40)	WHO AES	No	All	Yes	Most	Most
Timor Leste	National	WHO AES	No	All	Yes^¶¶¶^	Most	No
Vietnam	National and sentinel (8)	WHO AES, AMES****	Yes^††††^	All (AES); <15 yrs (AMES)	Yes	Most	Most

**Abbreviations:** AMES = Acute meningoencephalitis surveillance; AES = Acute encephalitis surveillance CSF = cerebrospinal fluid; NA = not available.

* Most = country reported testing specimens from ≥50% suspected JE cases. Some = country reported testing

^†^ JE virus transmission risk in well-defined, limited areas.

^§^ Torres Strait Islands and northern Cape York.

^¶^ Clinical evidence of non-encephalitic disease (acute febrile illness with headache, myalgia and/or rash) or encephalitic disease (e.g., focal neurologic disease, impaired level of consciousness, abnormal brain imaging study, abnormal encephalogram, and/or presence of pleocytosis in cerebrospinal fluid) plus definitive laboratory evidence of JE infection.

** Encephalitis and meningitis surveillance integrated for sentinel but not national surveillance program.

^††^ A clinical case was defined as a person of any age with an acute onset of fever and a change in mental status and/or a new onset of seizures (excluding simple febrile seizures) at any time of the year. A confirmed case was defined as a clinical case with a positive laboratory test specific for JE in serum, plasma, blood, CSF or tissue or that met the clinical case definition and was epidemiologically linked to a confirmed case (Chang YK, Chang HL, Wu HS, Chen KT. Epidemiological features of Japanese encephalitis in Taiwan from 2000 to 2014. Am J Trop Med Hyg 2017;382–8).

^§§^ Reported “Yes” but did not quantify percentage.

^¶¶^ Patients with encephalitis syndrome with laboratory-confirmed JE.

*** Encephalitis and meningitis surveillance integrated for national (but not sentinel) surveillance program.

^†††^ Febrile illness with neurologic symptoms (e.g., headache, meningeal signs, stupor, disorientation, coma, tremors, general paresis, hypertonia, loss of consciousness).

^§§§^ Reported “Yes” but did not quantify percentage. Also, reported that laboratory has not performed a JE diagnostic test on a human sample since 2014.

^¶¶¶^ Testing suspended because of reagent stockouts in 2016.

**** Five sentinel sites use AES and three use AMES case definition.

^††††^ At AMES sites.

During 2015, WHO received reports of 4,087 JE cases from 20 (83%) of 24 countries; 3,549 (87%) of these cases were reported from four countries (China [624 cases], India [1,620], Nepal [937], and Vietnam [368]). No other country reported more than 115 cases.

## Immunization Programs

Twelve (50%) of the 24 countries had a JE immunization program in 2016 (Table 2); 10 (42%) programs were implemented nationally or subnationally in all risk areas, and two (8%) were subnational and did not include all risk areas. Six countries used live attenuated vaccine, two used live recombinant vaccine, one used an inactivated Vero cell culture-derived vaccine, one used an inactivated mouse brain-derived vaccine,^††^ and two used multiple vaccine types.

**TABLE 2:** Characteristics of Japanese encephalitis (JE) immunization programs in countries with JE virus transmission risk, 2016

Country	JE immunization program	Strategy	Scheduled age to begin routine immunization	Vaccine used in national program
Australia*	All risk areas^†^	Routine	12 mos	JE-CV
Bangladesh	None	—	—	—
Bhutan	None	—	—	—
Brunei	None	—	—	—
Cambodia	National	Routine	9 mos	CD-JEV
Burma	None^§^	—	—	—
China	National^¶^	Routine	8 mos	CD-JEV
Taiwan	All areas	Routine	15 mos	MB
India	Subnational**	Routine	9–11 mos	CD-JEV
Indonesia	None^††^	—	—	—
Japan	National	Routine	6 mos	VC
Laos	National	Routine	9–11 mos	CD-JEV
Malaysia	Subnational^§§^	Routine	9 mos	JE-CV
Nepal	National	Routine	12 mos	CD-JEV
North Korea	None^¶¶^	—	—	—
Pakistan	None	—	—	—
Papua New Guinea	None	—	—	—
Philippines	None***	—	—	—
Russia*	None	—	—	—
Singapore	None^†††^	—	—	—
South Korea	National	Routine	12 mos	CD-JEV, MB, VC,
Sri Lanka	National	Routine	12 mos	CD-JEV
Thailand	National	Routine	12 mos	CD-JEV, JE-CV
Timor Leste	None	—	—	—
Vietnam	National	Routine	12 mos	MB

**Abbreviations:** CD-JEV = live attenuated JE vaccine; JE-CV = live recombinant JE vaccine; MB = inactivated, mouse brain-derived JE vaccine; VC = inactivated, Vero cell culture–derived JE vaccine.

* JE virus transmission risk in well-defined, limited areas.

^†^ Vaccination recommended for residents of the outer Torres Strait Islands or nonresidents living or working there for ≥30 days during the wet season.

^§^ Burma is planning a national JE vaccination campaign for 2017, followed by routine introduction.

^¶^ Excluding the provinces of Qinghai, Tibet, and Xinjiang, which do not have endemic transmission.

** JE vaccine included in 216 districts with endemic JE.

^††^ Indonesia will initiate JE vaccine campaign in Bali in 2017.

^§§^ In Sarawak state; in peninsular Malaysia and Sabah, vaccination is provided to children aged <15 years in the vicinity of an outbreak.

^¶¶^ North Korea conducted a JE vaccination campaign in 2016.

*** Philippines is planning a subnational JE vaccination campaign in 2018, followed by routine introduction nationally.

^†††^ Singapore made a decision not to introduce JE vaccine because only rare, sporadic human cases are reported in the country.

## Discussion

Since 2012, JE surveillance and immunization programs have expanded and improved. In 2016, 92% of countries with JE virus transmission risk conducted JE surveillance compared with 75% in 2012, and two countries that only conducted sentinel surveillance in 2012 were conducting surveillance nationally or subnationally in all risk areas in 2016 (*2*). The percentage of countries that had a JE immunization program increased slightly, from 46% in 2012 to 50% in 2016. Larger increases were reported in breadth of implementation: programs in 42% of countries were implemented nationally or in all risk areas compared with only 25% in 2012 (*2*). Several countries have transitioned from using mouse brain-derived vaccine to newer, less reactogenic vaccines with simpler dosing schedules, as recommended by WHO (*1*). Only two (8%) countries currently use mouse brain-derived vaccine (including one that uses multiple vaccine types), compared with five (21%) countries that used this vaccine in 2012.

The number of reported JE cases was approximately 60% lower in 2015 than in 2011, and there was a change in the proportions of reported cases by country. In 2011, China and India accounted for nearly 95% of JE cases reported to WHO (*2*), compared with only 55% in 2015. From 2011 to 2015, the number of cases reported by Nepal increased elevenfold from 75 to 937, and the number reported from Vietnam doubled from 183 to 368. However, because of substantial underreporting of cases, potential inconsistencies in reporting, or changes in surveillance practices, and the known year-to-year variability in intensity of JE virus transmission, the significance of changes based on surveillance data from these two time points is not known. However, JE vaccine impact assessments indicate immunization programs can result in substantial reductions in JE cases; if high coverage can be achieved and maintained in countries with endemic transmission, JE disease might be practically eliminated even while the virus remains in circulation (*8*).

JE surveillance has been established or strengthened during the last 4 years in several countries; since 2012, national surveillance programs were established in Brunei, North Korea, and Timor Leste, and expanded in India and Nepal. However, the need to enhance the quality of JE surveillance is recognized (*6*). More countries reported availability of laboratory diagnostic testing for suspected JE cases, and most report testing of both serum and CSF specimens, although the percentage of suspected JE cases for which testing is performed is unknown. Reported increases in diagnostic testing might in part be explained by support provided by the JE laboratory networks that were established in WHO's South-East Asia and Western Pacific regions during 2006–2008. WHO has developed a JE laboratory accreditation program, which includes proficiency testing, confirmatory testing, and other measures to ensure high quality laboratory testing.

Substantial progress has been made in establishing and strengthening JE immunization programs. During 2015–2016, Nepal’s JE immunization program expanded from a subnational to a national program after conduct of a catch-up campaign, and both Cambodia and Laos established national JE vaccination programs following catch-up campaigns in children aged <15 years. Burma, Indonesia, and the Philippines plan to introduce JE vaccine in late 2017 or early 2018. Progress has been aided by the availability of three WHO-prequalified JE vaccines; enhanced awareness of the importance of JE prevention and control; and increased commitment by governments, international organizations and nongovernmental organizations such as PATH, the Bill and Melinda Gates Foundation, and the GAVI Alliance.

Despite this progress, gaps and challenges remain, including incomplete case reporting and misclassification of cases. For example, the limited scope of surveillance in some countries results in incomplete case ascertainment, and data needed to improve suspected case classification to guide program expansion and laboratory capacity enhancement are insufficient. Immunization program monitoring data, such as the vaccination histories of JE cases, are often not collected. In addition, monitoring of vaccination coverage following JE vaccine introduction, critical for ensuring achievement of coverage targets, is often inadequate. Finally, more complete and accurate JE disease data are needed to estimate global burden.

The findings in this report are subject to at least two limitations. First, data were collected from self-administered surveys and might be susceptible to social desirability, recall, or other biases. Second, reported data might be incomplete.

Vaccination is the most effective strategy to prevent and control JE, and immunization has been demonstrated to reduce the economic burden of JE disease (*1*,*8*). In 2014, the countries of the WHO Western Pacific Region endorsed a goal to accelerate the control of JE by extending vaccination to all JE risk areas where incidence exceeds very low levels (*9*). Furthermore, countries in the WHO South-East Asia Region are developing a plan for accelerated control of JE by extending vaccination to all areas with any risk of JE transmission. WHO updated its JE vaccine position paper in 2015 (*1*) and produced a guidance document for measuring the effectiveness and impact of JE vaccination (*7*). Strengthened surveillance, continued commitment, and adequate resources for JE vaccination should help maintain progress toward prevention and control of JE.

**Summary**

**What is already known about this topic?**

Japanese encephalitis (JE) virus is a leading cause of encephalitis in Asia. The World Health Organization recommends integration of JE vaccination into national immunization schedules in all areas where the disease is a public health priority.

**What is added by this report?**

A review of surveillance and immunization program data in the 24 countries with JE virus transmission risk found that in 2016, 22 countries conducted at least some surveillance for JE, and 12 had implemented a JE immunization program. This represents substantial progress in JE prevention and control measures, but challenges remain, including incomplete case reporting, misclassification of cases, lack of immunization program monitoring data, and inadequate monitoring of JE vaccination coverage following vaccine introduction.

**What are the implications for public health practice?**

Strengthened surveillance, continued commitment, and adequate resources for JE vaccination should help maintain progress toward prevention and control of JE.

## Footnotes

* Prequalification by WHO is a process through which the quality, safety, and efficacy of medicinal products is assessed. Prequalified products meet specified requirements, and the associated manufacturing site(s) and contract research organization(s) are determined to be compliant with WHO standards. http://www.who.int/mediacentre/factsheets/fs278/en/.

† Surveys requested information on existence of a JE surveillance program, whether a JE case definition is used, integration of encephalitis and meningitis surveillance, age groups under surveillance, availability of laboratory confirmation of JE, existence of a JE immunization program, strategy used for JE immunization, scheduled aged groups for routine JE immunization, and types of JE vaccines used in the national immunization program.

§ The Joint Reporting Form is a standard questionnaire sent annually to all WHO countries, through which WHO and the United Nations Children’s Fund jointly collect immunization data.

¶ A survey was not administered to health officials from Taiwan. Data for Taiwan were obtained from published literature and the Taiwan CDC website.

** AMES (acute meningitis/encephalitis syndrome) is defined as an acute febrile illness that is clinically compatible with meningitis and/or encephalitis.

†† Mouse brain-derived JE vaccine is also used in Taiwan's JE immunization program.
