# Erratum: Vol. 66, No. 22

**DOI:** 10.15585/mmwr.mm6624a7

**Published:** 2017-06-23

In the report “Japanese Encephalitis Surveillance and Immunization — Asia and Western Pacific Regions, 2016,” on page 581, in the table “Characteristics of Japanese encephalitis (JE) surveillance in countries with JE virus transmission risk, 2016,” and on page 582, in the table “Characteristics of Japanese encephalitis (JE) immunization programs in countries with JE virus transmission risk, 2016,” Taiwan should have been indented beneath China. The corrected tables with added indents can be found at http://apps.who.int/iris/bitstream/10665/255639/1/WER9223.pdf.

